# Positive Psychology Themes in Interviews of Children With Atopic Dermatitis: Qualitative Study

**DOI:** 10.2196/38725

**Published:** 2022-09-14

**Authors:** Terry M Lou, Kenneth L Zhang, Noël C Slesinger, Michelle Taddeo, Eloisa Serrano, Wendy Smith Begolka, Korey Capozza, Amy S Paller, James W Griffith, Anna B Fishbein

**Affiliations:** 1 Department of Pediatrics Ann & Robert Lurie Children's Hospital Chicago, IL United States; 2 Division of Pediatric Allergy & Immunology Northwestern University Chicago, IL United States; 3 Department of Medical Social Sciences Northwestern University Feinberg School of Medicine Chicago, IL United States; 4 National Eczema Association Novato, CA United States; 5 Global Parents for Eczema Research Santa Barbara, CA United States; 6 Department of Dermatology Northwestern University Feinberg School of Medicine Chicago, IL United States; 7 Department of Pediatrics Northwestern University Feinberg School of Medicine Chicago, IL United States

**Keywords:** positive psychology, PERMA, positive emotion, engagement, relationships, meaning, and accomplishment, atopic dermatitis, pediatric, dermatology, children

## Abstract

**Background:**

Atopic dermatitis is a pruritic chronic condition associated with significant sleep disturbance, inattention, and sometimes behavioral problems. Enhancing resiliency in children with atopic dermatitis may promote coping strategies to improve quality of life. Positive psychology is one strategy that can be used to strengthen resiliency.

**Objective:**

Our objective was to identify positive psychology concepts mentioned by children with atopic dermatitis and their parent to inform strategies to strengthen resiliency in children with atopic dermatitis.

**Methods:**

A total of 20 patient-parent dyads were interviewed to share their experience with atopic dermatitis to help develop a novel psychologic intervention for atopic dermatitis. Patients were 8 to 17 years old and diagnosed with atopic dermatitis. Trained coders analyzed transcripts using a coding dictionary developed based on Seligman’s PERMA (positive emotion, engagement, relationships, meaning, and accomplishment) model of positive psychology. The frequency of unprompted mentions of PERMA themes and relevant quotations was captured. Transcripts were also separately coded for resiliency, which is the ultimate goal of PERMA.

**Results:**

Positive psychology concepts were mentioned by 100% (20/20) of children and 95% (19/20) of parents. Engagement and relationships, both negative and positive aspects, were the most common unprompted PERMA themes mentioned by children (14/20, 70%) and parents (13/20, 65%). Emotion elicited the most negative comments from children (19/20, 95%) and parents (17/20, 85%). When analyzed for resiliency, 8 participants were identified with at least one resiliency code. On average, participants with a resiliency code mentioned PERMA concepts 9.1 (SD 4.7) times compared to those who mentioned none (mean 5.9, SD 4.6) (*P*=.14). When participants were stratified by disease severity, on average, more positive psychology concepts were mentioned by patients with mild atopic dermatitis (mean 13, SD 3.0) than those with moderate symptoms (mean 6.2, SD 4.9) or severe symptoms (mean 6.1, SD 4.0) (*P*=.03).

**Conclusions:**

Among PERMA themes, engagement and relationships are the two most commonly mentioned categories for children with atopic dermatitis. Strategies targeting PERMA such as affirmations and positive reframing may improve psychosocial well-being and resiliency in pediatric atopic dermatitis. Future directions will look at incorporating “positive medicine” into atopic dermatitis treatment to not only relieve symptoms but also strengthen positive aspects of life.

## Introduction

### Background

Atopic dermatitis, also known as eczema, is a common pediatric chronic disease characterized by severe itch that is prevalent in the pediatric population. About 10% to 20% of children in the United States have atopic dermatitis, which can cause significant sleep disturbance, inattention, and sometimes behavioral problems [1,2]. Patients with atopic dermatitis are generally not severely ill and rarely require hospitalization. However, they experience significant psychologic stress and poor quality of life. Some examples include difficulty participating in sports due to sweat-induced itch, stigma with potentially disfiguring visible lesions, and psychologic repercussions of chronic sleep disturbance, such as depression and anxiety [3]. Enhancing resiliency in children with atopic dermatitis may promote coping strategies to improve itch, attention, and behavioral problems [4]. One study that included adult patients with atopic dermatitis looked at the consistent use of an internet-based positive psychology intervention and demonstrated improved well-being [5].

One strategy for strengthening resiliency in children is via positive psychology. This emerging field of study is in contrast to traditional problem-based psychology, which focuses on the deficits in one’s life, such as how mental health diagnoses negatively impact one’s well-being. Positive psychology looks at what makes the individual feel well and considers ways to enhance their well-being. High levels of feasibility and acceptability of positive psychology interventions make this a relevant approach for atopic dermatitis, specifically to translate positive psychology interventions to enhance well-being and health outcomes [6]. The field of positive psychology presents unique opportunities to enhance the well-being of children with chronic disease. This is particularly important as the prevalence of pediatric chronic disease has increased in the past decades [7].

### Goal of This Study

Our objective was to analyze interview transcripts from child-parent dyads about atopic dermatitis using the PERMA (positive emotion, engagement, relationships, meaning, and accomplishment) model to determine which positive psychology themes were mentioned and whether there were areas of improvement for the well-being of children with atopic dermatitis using a positive psychology approach.

## Methods

### Recruitment

Patient-parent dyads of children aged 8 to 17 years old with atopic dermatitis were identified as a convenience sample recruited from the dermatology or allergy clinic at the Ann & Robert Lurie Children’s Hospital of Chicago. Inclusion criteria included children with physician-diagnosed atopic dermatitis currently receiving treatment in clinic. Disease severity was assessed by an allergist or dermatologist. Exclusion criteria included non–English-speaking parent-child dyads, history of intellectual disability or psychosis, and uncontrolled atopic dermatitis. A total of 49 dyads were screened for recruitment, 24 pairs were eligible for the study, 23 patients were enrolled, and 3 dropped out, with 20 patient-parent dyads ultimately completing the study. Participants were interviewed to share their personal experiences with atopic dermatitis to develop a psychologic intervention. Positive psychology themes emerged during the analysis, which were further explored in this study.

Trained coders analyzed the qualitative data using a coding dictionary developed based on Seligman’s PERMA model of positive psychology (Multimedia Appendix 1). The PERMA model breaks down positive psychology into 5 categories that may be targeted to improve well-being: positive emotion, engagement, relationships, meaning, and accomplishment. Two coders independently reviewed all interview transcripts, coding for mentions of PERMA themes or the lack thereof. Coders then had a discussion to reconciliate differences in codes. Any persistent discrepancies were resolved by a third party. Transcripts were also analyzed by a separate pair of coders using a constant comparative approach. The emergent themes included resiliency, which was investigated for this study.

### Ethics Approval

Approval was granted by the Institutional Review Board of Ann & Robert Lurie Children's Hospital of Chicago (#IRB 2019-2560).

## Results

Among the 20 child participants, the average age was 12 (SD 1.9) years. Of the participants, 9 (45%) were male, 11 (55%) were female, 7 (35%) identified as White, 7 (35%) as Black/African American, 3 (15%) as Latino or Latina, and 3 (15%) as Asian. Disease severity was assessed by a clinician global assessment or the exam-based Eczema Area and Severity Index: mild (n=3, 15%), moderate (n=9, 45%), and severe (n=8, 40%). At the time of the interview, all patients were on topical prescription therapies, and 7 (35%) participants were on oral or subcutaneous systemic therapy for atopic dermatitis. In terms of other chronic allergic diseases, 11 (55%) participants had asthma, 6 (30%) had allergic rhinitis, and 6 (30%) had food allergies.

Unprompted mentions of engagement and relationships were the most common PERMA themes raised by children (n=14, 70%) and parents (n=13, 65%) (Table 1). Interestingly, children and parents equally brought up negative and positive aspects of engagement and relationships due to eczema. Emotion elicited the most negative comments from children (n=19, 95%) and parents (n=17, 85%).

We also stratified participants by disease severity to analyze the frequency of positive psychology concepts mentioned. Positive psychology concepts were mentioned more frequently by patients with mild atopic dermatitis (concepts: mean 13, SD 3; patients: n=3, 15%) versus those with moderate atopic dermatitis (concepts: mean 6.2, SD 4.9; patients: n=9, 45%) and those with severe atopic dermatitis (concepts: mean 6.1, SD 4; patients: n=8, 40%) (*P*=.03).

Transcripts were also analyzed for resiliency codes. Eight participants had at least one resiliency code. A sample of resiliency quotes is summarized in Textbox 1. Participants with a resiliency code mentioned 9.1 (SD 4.7) positive psychology concepts on average throughout their interview, whereas participants without a resiliency code mentioned 5.9 (SD 4.6) positive psychology concepts on average (*P*=.14).

**Table 1:** Positive psychology examples of PERMA (positive emotion, engagement, relationships, meaning, and accomplishment) themes and counterexamples mentioned unprompted in interviews.

PERMA category			Participants who mentioned the concept, n (%)				Example
			Parent		Child		
Positive emotion			3 (15)		9 (45)		“So it’s actually comforting…to be at home.” [Child]
Negative emotion			17 (85)		19 (95)		“[The itching] is frustrating for him that he can’t stop.” [Parent]
Engagement			10 (50)		18 (90)		“I don’t want to let [the itch] keep me from the stuff so I keep doing the things.” [Child]
Lack of engagement			9 (45)		17 (85)		“If I’m trying to do something and I feel itchy, it’s hard to do that thing ‘cause it’s distracting.” [Child]
**Unhindered relationships**			11 (55)		15 (75)		
	Adults	9 (45)		12 (60)		Interviewer: “How do you talk about [your eczema] with adults?” Participant [child]: “As if I’m talking to my friends, it’s not that big of a deal.”	
	Peers	5 (25)		13 (65)		“If somebody new that doesn’t know her would ask her about her eczema ‘what is that?’… her friends will say something ‘it’s eczema.’ So she’s got a good support group.” [Parent]	
	Other	2 (10)		11 (55)		“Most people aren’t going to say anything [about your eczema] but if they do, just ignore them. It doesn’t matter what they say.” [Child]	
**Hindered relationships**			11 (55)		16 (80)		
	Adults	5 (25)		8 (40)		Interviewer: “Do you ever avoid meeting new adults because of your itching?” Participant [child]: “If they ask too many questions, then yeah.”	
	Peers	5 (25)		10 (50)		“She’s gotten made fun of [because of her eczema].” [Parent]	
	Other	3 (15)		7 (35)		“I just try to avoid the subject [of my eczema]…I don’t think they understand.” [Child]	
Meaning			4 (20)		6 (30)		“I’m not really scared of having to itch…it doesn’t matter whether it’s here or not.” [Child]
Lack of meaning			4 (20)		2 (10)		“She’ll scream and say why do I have to be born this way, I hate my skin.” [Parent]
Accomplishment			8 (40)		7 (35)		“he handles [the itch] all by himself. I actually didn’t realize it gave him a lot of trouble…[he takes] care of it himself.” [Parent]
Lack of accomplishment			4 (20)		5 (25)		“I can’t really do anything about [my frustration due to eczema].” [Child]

**Sample of resiliency quotes from parent and child transcripts.**

**Parent**

- “They’re not self-conscious about [their eczema]…I believe that’s because there are other children in the school system that have eczema.”
- “She’s becoming independent, which is good, she likes to do [her eczema care regimen] herself…she does a great job”
- “I try to tell her…‘[child], I think that overall life is gonna be somewhat easier for you because you’ve had to learn how to deal with this, so I think some things are gonna come a lot easier to you’”
- “No one made fun of me [for my eczema]. So, I’m thinking maybe people just understand and other people have their own issues too…he hasn’t complained about it”

**Child**

- “If someone’s making fun of your skin just don’t be friends with them.”
- “[I] think about like long-term effects, just thinking about like oh, right now it would be best if I just don’t itch. Like it’s great if my skin looks clean now just focus on it right now, don’t like worry about how it’ll look like a month from now.”
- “Especially the kids who I talk to, they get it because they, everybody has a problem, nobody is perfect so when I talk about it I just say…I have eczema blah blah blah and then they don’t care that much afterwards”
- “We do all this stuff to help [my eczema], I know it’s not going to stay bad forever”
- “I know [the itch] it’s gonna to come back but it doesn’t worry me too much”
- “I’ve had eczema severely my whole life so like just if you do it in front of people nobody really cares that much because eczema is the thing that tons of people have so…I just tell them that it’s a normal thing”
- “I would tell [people who ask about my itching] that it gets better as life goes on if you just find the right thing”
- “I feel like I don’t like want to let [my eczema] keep me from the stuff so I keep doing the things”

## Discussion

### Principal Findings

Our small but diverse sample of children with atopic dermatitis frequently mentioned positive psychology concepts in qualitative interviews about their personal experience with atopic dermatitis. Children with severe disease were less likely to mention positive psychology concepts. Across all patients, the concepts of relationships and engagement were most frequently mentioned. Previous work in pediatric chronic disease shows similar findings that the concepts of relationships and engagement are consistently impacted.

With regard to relationships, children with chronic disease report difficulty maintaining relationships with family and friends [8,9]. They must also deal with concerns including how to share their diagnosis with others [10] and how to cope with unwanted attention [11]. Atopic dermatitis itself can become a source of conflict, especially given the stigma around having a visible skin condition. Children with chronic conditions need a supportive community to cope with the stress of their disease and management [12]. As several patients and parents mentioned, a supportive home environment and group of friends helped boost relationship building for them. Encouraging positive relationships, even by simple questions in clinic about close friends or family members, should be considered by providers treating atopic dermatitis.

With regard to engagement, children with chronic disease report worse school experiences and less participation in extracurricular activities compared to their healthy counterparts [8,13,14]. For many, their disease causes significant limitation of normal functions [15]. As an example, children with atopic dermatitis might limit physical activity, as they frequently report sweat-related itch and skin pain when sports equipment rubs against their skin. These children may benefit from additional support to help them engage with activities despite these physical limitations or pivot to activities with less physical discomfort to increase levels of engagement. Providers should consider querying about enjoyable activities.

While all PERMA categories are valuable to cultivate in patients with atopic dermatitis, our study identified positive emotion as the most needed area to cultivate. This is not surprising as previous work in atopic dermatitis demonstrated a correlation with negative emotion, poor quality of life, and disease severity [16]. Children with chronic conditions often experience significant negative emotions related to their disease, including but not limited to functional impairment, treatment burden, and acute as well as long-term stress [17]. Positive psychology alone cannot eliminate these negative emotions, but positive psychology interventions can enhance positive emotions, with favorable outcomes including enhanced resiliency and coping [18]. Positive emotion interventions include exercising gratitude and affirmations, which can be elicited by the parent or provider and also via several apps [19]. Cultivating gratitude through apps or physically writing letters of gratitude is easy to learn and can be frequently practiced to help strengthen positive emotion.

There is potential utility in adopting positive psychology interventions for children with atopic dermatitis, particularly in populations with severe disease to inspire them to build resilience and improve psychosocial health that could lead to improved health outcomes through, for example, less rumination and medication adherence.

### Limitations and Conclusion

Limitations to our study include the small sample size, the exclusion of non–English-speaking patients, and interview questions that were not specific to positive psychology. Interview questions tended to focus on how atopic dermatitis has negatively impacted patients’ lives, without specifically soliciting more information on how positive psychology concepts could or are improving participant well-being (Multimedia Appendix 2). We hope to encourage further research on the application of positive psychology in pediatric atopic dermatitis and other pediatric chronic diseases.

## Appendix

Multimedia Appendix 1 Coding dictionary, developed based on Seligman’s PERMA (positive emotion, engagement, relationships, meaning, and accomplishment) model of positive psychology.

Multimedia Appendix 2 Interview questions.

## Abbreviations

PERMA: positive emotion, engagement, relationships, meaning, and accomplishment
