# Supplementary material for: Positive Psychology Themes in Interviews of Children With Atopic Dermatitis: Qualitative Study
Source: JMIR Pediatr Parent. 2022 Sep 14;5(3):e38725. doi: 10.2196/38725 (PMC9520397; doi:10.2196/38725)
Supplement: Multimedia Appendix 1 [file pediatrics_v5i3e38725_app1.docx]

Appendix 1. Coding dictionary, developed based on Seligman’s PERMA model of positive psychology

|  | + | - |
| --- | --- | --- |
| Positive Emotions | Positive Emotion   - Positive emotion: happiness, joy, contentment, compassion, gratitude - Optimism: choosing not to worry about something anymore - NOT simply reducing negative emotion (more psychotherapy versus positive psychology) | Negative Emotion   - Negative emotion: anxiety/fear, embarrassment (self-conscious), shame/guilt, jealousy, anger - UNLESS this is followed with an addendum that they are not dwelling in it - NOT physical symptoms like pain, etc. |
| Engagement | Engagement   - Participating in activities where ‘time flies by, absorbed’ - Still able to participate in engaging activities despite their symptoms/disease - Distracting themselves from itch by actively engaging in another activity - DO NOT CODE absentmindedly distracting themselves - DO NOT CODE if kid does not avoid ‘things’ | Lack of engagement   - Symptom/disease leads to less engagement (including distracted, can’t focus, etc.) |
| Relationship | Unhindered relationship   - Talking about eczema doesn’t bother them - Able to rely on caregivers for treatment and support - Sense of belonging with other kids with eczema | Hindered relationship   - Don’t want to talk about their eczema or their feelings about eczema - Loneliness |
| Meaning | Meaning+   - Acceptance of diagnosis that their eczema does not define them, not through avoidance - Cause bigger than me: religion, spirituality, science, volunteering/helping others | Lack of thought   - Unaccepting of diagnosis, ‘why me,’ ‘life’s not fair’ - Only talk about disease in necessity (focusing on physical symptoms, medicines, appointments, etc.) |
| Accomplishment | Accomplishment   - Goals, ambition - Achieving small daily tasks - Mastery/competence, specifically disease management, i.e.: I take medicine and it feels better | Lack of willpower   - Fail to achieve your goals/daily tasks - Poor disease self-management - Powerlessness, ‘things happen to me,’ ‘life is beyond control’ |
