# Supplementary material for: Positive Psychology Themes in Interviews of Children With Atopic Dermatitis: Qualitative Study
Source: JMIR Pediatr Parent. 2022 Sep 14;5(3):e38725. doi: 10.2196/38725 (PMC9520397; doi:10.2196/38725)
Supplement: Multimedia Appendix 2 [file pediatrics_v5i3e38725_app2.docx]

Appendix 2. Interview questions

1. Patient and parent understanding of atopic dermatitis
   1. First, can you tell me what you know about eczema? You may have also heard it called atopic dermatitis. If needed, education can be provided from the background section.

Possible follow-up questions:

- What do you think causes eczema?
- Why do you think some people get eczema?
- How many people do you think have eczema?
- What are some of the symptoms of your eczema?
- What things help with the itching?
- What things make the itching worse?
  1. Is there anything else that is unclear about eczema or caring for your eczema?
  2. What do you do if you do have a question about your eczema?

Optional reminder: If you do have questions or don’t know what to do, you can always ask your doctor. It can also be helpful when a question comes up to write it down and bring the list of questions to your next appointment. The therapist can also reinforce communication through the electronic medical record if the patient has the capability at this time.

- 1. Provide empathy around feelings of unfairness or frustration.

1. Adherence to medication
   1. Now I would like to know a little bit about the different medications you use for your eczema…
   2. Can you tell me what creams or medications you use for your eczema? These can be creams or pills you got from your doctor and things you may get from the store.
   3. Can you tell me when you are supposed to use those creams and medications that you just told me about? If patient is struggling, ask them to tell you what medications they should be using each day and if medications change by day.
   4. Everyone may miss some medications sometimes or forget to put on creams. So, if you had to guess, how often do you think you remember to use your creams and medications the way the doctor told you to between none or 0% of the time and 100% or all of the time?

Patients may have difficulty quantifying this amount, in this case, you can use prompts, such as: Do you think it’s more or less than half the time? If you have used a measure of medication adherence and the response differs significantly from this, also ask about the discrepancy at this time.

1. Strategies used to cope with itch
   1. Sometimes people will try different things to help with their itch, Is there anything that helps when you itch?

Note: For each item mentioned, probe with the following:

- How well does that work for you?
- How often does that help?
- Are there times when you can’t use [insert strategy] or it does not work?
  1. Is there anything that makes the itch worse?

Note: For each item mentioned, probe with the following:

- What do you do when that happens?
- Are there any changes you have made that have helped?
- If the patient or family talks about avoiding certain things (e.g. certain clothes or activities): Are there times when you can’t avoid those things? How do you handle that?

1. Social concerns

A lot of kids with eczema have talked about have trouble at school or with friends because of their eczema…

- 1. Has anyone ever made fun of you because of your eczema? If so, can you tell me a little bit about it?
  2. How do you talk to other children about your eczema? What about other adults?

Follow-up questions:

- What do you say to people about your eczema?
- If they avoid talking about their eczema: Why don’t you talk about it with others?
- What do you say to people when they ask?
  1. Are there any things you avoid because you don’t want others to you’re your eczema? Examples, such as certain clothes or sleepovers can be provided here.

1. Mood and anxiety related to atopic dermatitis

Now I want to ask you about how you feel when you itch.

- 1. How do you feel when you itch? If patient is struggling with an answer or says they have no feelings, that therapist can probe with: Some kids have told us they feel frustrated or angry, sometimes sad or worried, has anything like this ever happened to you?
  2. How do you feel about having eczema?

Possible follow-up questions:

- Do you know anyone else with eczema?
- The assessor can also probe concerns noted above and feelings around these areas at this time.
  1. When you aren’t itchy, do you ever worry about the itch coming back? If the patient says yes, ask about what they do when they worry about this.

1. Functional Impairment
   1. Sometimes it can be difficult for kids with eczema to do certain things because of their eczema, Is there anything that is harder for you to because of itch? Assessor can provide some examples if needed such as sports, outside activities, concentrating on schoolwork or in class, being unoccupied.
2. Sleep concerns

Now, I want to ask you some questions about your sleep.

- 1. Do you ever have a hard time falling asleep because of the itch?

Follow-up questions:

- Is it hard to fall asleep because you are itching?
- Is it hard to fall asleep even when you are not itchy?
- Do you ever worry about itch coming back when you are trying to fall asleep?
- What do you do when you can’t fall asleep?
  1. Do you ever wake up at night because of itching?

Follow-up: What do you do when this happens?
